# Physiological and Pathological Roles in Human Adrenal of the Glomeruli-Defining Matrix Protein NPNT (Nephronectin)

**DOI:** 10.1161/HYPERTENSIONAHA.117.09156

**Published:** 2017-05-10

**Authors:** Ada Ee Der Teo, Sumedha Garg, Timothy Isaac Johnson, Wanfeng Zhao, Junhua Zhou, Celso Enrique Gomez-Sanchez, Mark Gurnell, Morris Jonathan Brown

**Affiliations:** From the Clinical Pharmacology Unit, Centre for Clinical Investigation, Addenbrooke’s Hospital (A.E.D.T., S.G., J.Z., M.J.B.), Tissue Bank, Department of Histopathology, Addenbrooke’s Hospital (W.Z.), NIHR Cambridge Biomedical Research Centre, Addenbrooke’s Hospital (M.G.), MRC Cancer Unit, Hutchison/MRC Research Centre (T.I.J.), and Metabolic Research Laboratories, Wellcome Trust-MRC Institute of Metabolic Science (M.G.), University of Cambridge, United Kingdom; Centre for Clinical Pharmacology, William Harvey Research Institute, Barts and the London School of Medicine & Dentistry, Queen Mary University of London, United Kingdom (J.Z., M.J.B.); Division of Endocrinology, Department of Medicine, The University of Mississippi Medical Centre, Jackson (C.E.G.-S.); and Research and Medicine Services, G.V. (Sonny) Montgomery VA Medical Centre, Jackson, MS (C.E.G.-S.).

**Keywords:** adenoma, aldosterone, extracellular matrix, hypertension, Wnt signaling pathway

## Abstract

Primary aldosteronism is a common cause of hypertension, which becomes refractory if undiagnosed, but potentially curable when caused by an aldosterone-producing adenoma (APA). The discovery of somatic mutations and differences in clinical presentations led to recognition of small but common zona glomerulosa (ZG)–like adenomas, distinct from classical large zona fasciculata–like adenomas. The inverse correlation between APA size and aldosterone synthase expression prompted us to undertake a systematic study of genotype–phenotype relationships. After a microarray comparing tumor subtypes, in which *NPNT* (*nephronectin*) was the most highly (>12-fold) upregulated gene in ZG-like APAs, we aimed to determine its role in physiological and pathological aldosterone production. NPNT was identified by immunohistochemistry as a secreted matrix protein expressed exclusively around aldosterone-producing glomeruli in normal adrenal ZG and in aldosterone-dense ZG-like APAs; the highest expression was in ZG-like APAs with gain-of-function *CTNNB1* mutations, whose removal cured hypertension in our patients. NPNT was absent from normal zona fasciculata, zona fasciculata–like APAs, and ZG adjacent to an APA. NPNT production was regulated by canonical Wnt pathway, and *NPNT* overexpression or silencing increased or reduced aldosterone, respectively. NPNT was proadhesive in primary adrenal and APA cells but antiadhesive and antiapoptotic in immortalized adrenocortical cells. The discovery of *NPNT* in the adrenal helped recognition of a common subtype of APAs and a pathway by which Wnt regulates aldosterone production. We propose that this arises through NPNT’s binding to cell-surface integrins, stimulating cell–cell contact within glomeruli, which define ZG. Therefore, NPNT or its cognate integrin could present a novel therapeutic target.

## Abstract 2

Supplemental Digital Content is available in the text.

Five percent to 13% of all hypertension and 20% of resistant hypertension can be attributed to primary aldosteronism, of which unilateral aldosterone-producing adenoma (APA) is the most common curable cause.^1,2^ Early detection of APA is important because of significant increases in cardiovascular morbidity and mortality: congestive cardiac failure and ischemic heart disease are 2 to 5 times more prevalent,^3^ with an increase in 14-year mortality in these patients compared with matched patients with essential hypertension.^4^ Whether this additional risk is due directly to aldosterone excess, independent of high blood pressure, or reflects greater average duration of hypertension before diagnosis, clinical outcome is considered to benefit from prompt recognition and removal of APAs.^5^

Over the past decade, new molecular stratifications have enabled the recognition of a group of smaller zona glomerulosa (ZG)–like APAs.^6–8^ Compared with the classical large lipid-laden zona fasciculata (ZF)–like APA with mutations in inward rectifier potassium channel 4 (*KCNJ5*),^6^ not only is this ZG-like subtype of APA histologically and biochemically different, it also harbors hallmark somatic mutations in genes encoding a subunit of the voltage-gated calcium channel (*CACNA1D*),^8^ Na^+^/K^+^-ATPase (*ATP1A1*),^8,9^ Ca^2+^-ATPase (*ATP2B3*),^9^ or the Wnt pathway mediator β-catenin (*CTNNB1*).^10^ Biochemically, these smaller ZG-like APAs have a higher capacity for aldosterone production; semiquantitative analysis of immunohistochemical staining has revealed that CYP11B2 score is inversely correlated with tumor size and volume.^11,12^ In addition, ZG-like APAs are more responsive to angiotensin II, with higher levels of type 1 angiotensin II receptor mRNA.^13,14^ However, because of their small size, they are readily overlooked on cross-sectional imaging because computed tomography is unable to reliably detect adrenal tumors <5 to 6 mm.^15^ We hypothesized that specific gene products are responsible for the increased hormone production in these aldosterone-dense APAs and can potentially be used as a diagnostic biomarker when imaging proves inconclusive.

In seeking transcriptomic evidence to identify possible pathways of aldosterone production specific to the more compact, aldosterone synthase–dense APAs, we compared *CACNA1D/ATP1A1*-mutant with *KCNJ5*-mutant APAs.^8^ Extracellular matrix (ECM) gene *NPNT* (*nephronectin*) was found to be the most upregulated, by 12-fold, in the former and confirmed a categorical difference between APA genotypes by immunohistochemistry. Discovered in the kidney in 2001,^16^ *NPNT* was recently found to be a downstream Wnt target in the epidermis.^17^ This may be relevant in the adrenal, where normal adrenocortical development and steroidogenic activity of the ZG are dependent on the canonical Wnt pathway.^18^

Further examination of NPNT’s distribution presented in this article led us to hypothesize a key role in bringing ZG cells together to form functional units for aldosterone production through intercellular communication. This is supported by the observation, in rat ZG cells, of numerous tight junctions likely to be important in the establishment of electrical coupling,^19^ and the more recent discovery, only in intact adrenal slices, of oscillating membrane potentials regulating aldosterone secretion.^20^

In this study, we have found that *NPNT* is directly involved in the physiological secretion of aldosterone in the adrenal. By using both cell lines and primary human adrenal cells, we have also uncovered a previously unknown role of *NPNT* in adhesion and cell survival.

## Methods

### Human Subjects

Human adrenal tissues from patients who underwent adrenalectomy after being diagnosed with unilateral APA or pheochromocytoma were obtained from Cambridge University Hospitals’ Human Research Tissue Bank postsurgery at Addenbrooke’s Hospital, Cambridge, United Kingdom. All tissues were obtained with approval from the Cambridgeshire Research Ethics Committee with written informed consent before surgery. Further details are provided in the online-only Data Supplement and clinical features in Table S1 in the online-only Data Supplement.

### Cell Culture

Human adrenocortical carcinoma H295R cells were obtained from the American Type Culture Collection (ATCC CRL-2128), and grown in DMEM/Nutrient F-12 Ham supplemented with 10% FBS, 100 U penicillin, 0.1 mg/mL streptomycin, 0.4 mmol/L l-glutamine, and insulin-transferrin-selenium at 37°C in 5% CO_2_. Human embryonic kidney cells were obtained from the American Type Culture Collection (ATCC CRL-1573) and grown in DMEM supplemented with 10% FBS.

### Gene Overexpression and Silencing

Gene overexpression was performed using lipid-mediated cell transfection lipofectamine 3000 (Thermo Fisher), whereas gene silencing was achieved using DharmaFECT 1 lipid transfection reagent (Dharmacon), both according to manufacturer’s instructions. Cells were harvested for analysis of mRNA and protein expression after 48 hours. Further details are provided in the online-only Data Supplement.

### RNA Extraction, Reverse Transcription, and Quantitative Real-Time Polymerase Chain Reaction

Fifty to 100 mg of tissue or 1×10^5^ cells were used for each RNA extraction. Further details are provided in the online-only Data Supplement. Quantitative real-time polymerase chain reaction was performed using TaqMan ABI probes (Applied Biosystems) for *NPNT* (Hs01568126), *ITGB1* (Hs00559595), and *BCL2* (Hs00608023). *CYP11B2* expression was quantified using custom-made TaqMan probes (Invitrogen) previously validated for specificity.^21^

### Immunohistochemistry

Immunohistochemistry was performed using the peroxidase–antiperoxidase method on fresh frozen human tissue. In cases where fresh frozen tissue was unavailable, immunohistochemistry was performed on formalin-fixed, paraffin-embedded adrenal sections (4 μm) using an automated immunostainer with cover tile technology (Bond-III system; Leica Biosystems). NPNT antibody (HPA003711, Sigma; 1:50 dilution) and CYP11B2 antibody (custom mouse antihuman antibody from Dr Celso E. Gomez-Sanchez)^22^ were used as the primary antibodies. Further details are provided in the online-only Data Supplement.

### Aldosterone Measurement

Supernatant from cultured cells was used for aldosterone quantification using the homogenous time resolved fluorescence assay (Cisbio assays) based on the fluorescence resonance energy transfer technology, according to manufacturer’s instructions. The final fluorescence readout was conducted using a Pherastar FS microplate reader (BMG Labtech). Aldosterone concentrations were then normalized to total cell protein, quantified by the bicinchoninic acid protein assay (Pierce Biotechnology).

### Firefly/Renilla Luciferase Assay

To measure the activity of Wnt transcriptional complex T-cell factor/lymphoid enhancer factor (TCF/LEF), firefly luciferase and renilla luciferase activities were measured 48 hours after cotransfection with the Dual-Glo Luciferase Assay System (Promega) and normalized to the empty pCMV6 vector as described in the manufacturer’s protocol. Canonical Wnt signaling was quantified using the Cignal TCF/LEF reporter (luc) kit (SABiosciences).

### Cell Confluency and Cytotoxicity Assay

To measure changes in H295R cell confluency post-*NPNT* silencing, time-lapsed images were obtained using an Incucyte system (Essen BioScience). To differentiate changes in cell proliferation from cytotoxicity, cell-impermeant cyanine dimer nucleic acid stain YOYO-1 (Y3601; Life Technologies) was used. Further details are provided in the online-only Data Supplement.

### Annexin V–Propidium Iodide Dual Stain

To assess apoptosis over time, cells were double labeled with annexin V-APC (550474; BD Pharmingen) and propidium iodide (Sigma). After silencing, adherent cells were trypsinized, added to any detached cells in the supernatant as previously described,^23^ stained with annexin V–propidium iodide, and analyzed with the FACSCanto II flow cytometer (Becton-Dickinson). Further details are provided in the online-only Data Supplement.

### Xcelligence Cell Impedance Measurement and Hoechst Stain Assay

To evaluate changes in adhesion in response to NPNT, wells of an E-Plate 16 (ACEA Biosciences) were precoated with PBS, or 10 μg/mL of BSA, NPNT, or laminin for 1 hour at 37°C as previously described.^24^ Cell adhesion was also measured by Hoechst dye quantification of cells remaining on precoated wells post-wash. Further details are provided in the online-only Data Supplement.

### Proteins and Chemicals

Proteins used in this study were BSA (A9576; Sigma), NPNT (4298-NP-050; R&D systems), fibronectin (FC010; Millipore), and laminin (AG56P; Millipore). The selective porcupine inhibitor, LGK-974 (1 μmol/L; Selleck Chemicals), as used previously,^25^ was used to analyze the effect of blocking all Wnt secretion.

### Statistical Analysis

Results are expressed as mean values with SEM and compared using the 2-sided Student *t* test or by 1-way ANOVA followed by Tukey post hoc test. Significance level of *P*<0.05 was considered to indicate statistical significance. Statistical analysis was performed using Graphpad Prism (Graphpad Software).

## Results

### *NPNT* Is Selectively Expressed in the Subtype of Smaller ZG-Like APAs and Able to Distinguish Between the 2 APA Classes

**Figure 1.**
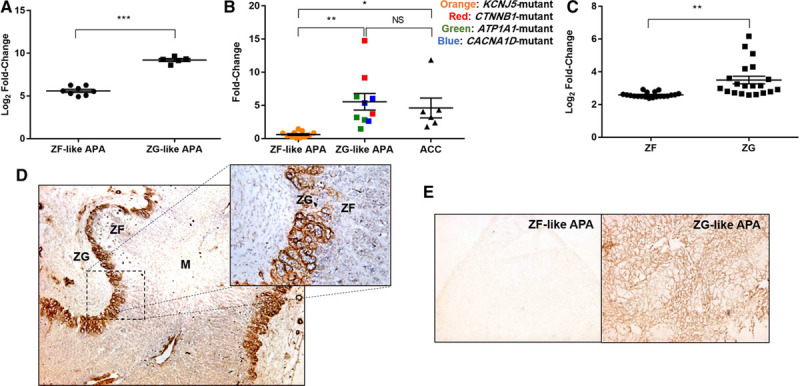
*NPNT* (*nephronectin*) is selectively expressed in normal adrenal zona glomerulosa (ZG), ZG-like aldosterone-producing adenomas (APAs), and adrenocortical carcinomas (ACCs). **A**, Microarray analysis of *NPNT*, comparing 8 zona fasciculata (ZF)-like adenomas with 5 ZG-like adenomas. **B**, Quantitative real-time polymerase chain reaction of *NPNT*, on mRNA extracted from 11 ZF-like APAs and 10 ZG-like APAs differentiated based on their genotypic mutations, as well as 6 ACCs. **C**, Microarray analysis of *NPNT*, comparing 20 paired ZF and ZG (each pair from the same patient), isolated via laser capture microdissection. **D**, Representative immunohistochemistry of NPNT showing selective extracellular localization in the ZG of adrenal adjacent to a pheochromocytoma (4× magnification; inset: 20× magnification). **E**, Representative immunohistochemistry of NPNT comparing staining between ZF-like APA and ZG-like APA mounted on the same slide (4× magnification). In (**A**)–(**C**), bars represent mean expression per group±SEM. Statistical analyses were conducted by Student *t* test (**A** and **C**) or 1-way ANOVA followed by Tukey post hoc test (**B**). *P<0.05; **P<0.005; ***P<0.0005. M indicates medulla; and NS, not significant.

Microarray analysis revealed *NPNT* to be 12.2-fold upregulated in the smaller ZG-like APAs with higher aldosterone synthetic capacity (Figure 1A). Validation with quantitative real-time polymerase chain reaction revealed a 9-fold difference; both ZG-like APAs and adrenocortical carcinomas (ACCs) had significantly elevated levels of *NPNT* compared with the ZF-like APAs (Figure 1B). The 2 APAs with the highest levels of *NPNT* harbored gain-of-function mutations in the Wnt gene *CTNNB1*.

In a further microarray comparing the ZG and ZF of 20 human adrenals isolated via laser capture microdissection,^26^ *NPNT* was on average 2-fold more highly expressed in the outer, aldosterone-producing zone of the ZG (Figure 1C). This selective expression was evident in protein immunohistochemistry, in which NPNT staining is localized exclusively to the ZG (Figure 1D; Figure S1). When mounted on the same slide, NPNT also makes it easy to differentiate between the 2 APA subtypes as there is negligible staining in ZF-like APAs (Figure 1E; Figure S2). In all cases, NPNT expression appeared extracellular and strikingly periglomerular, surrounding clusters of cells.

### *NPNT* Expression Corresponds to Aldosterone Synthase *CYP11B2* Expression

**Figure 2.**
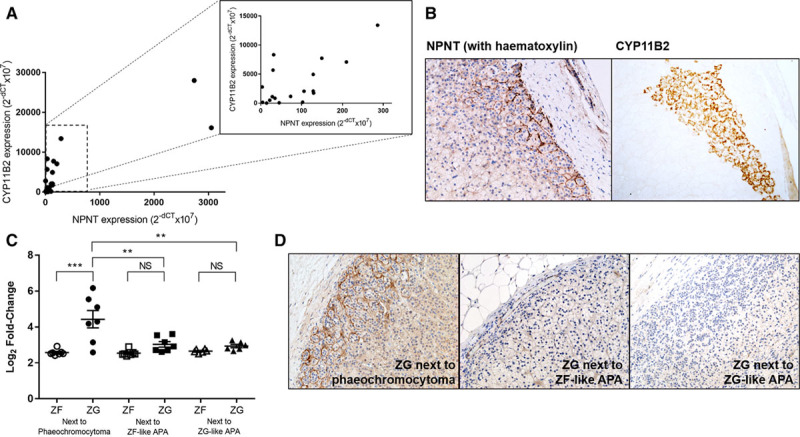
*NPNT* (*nephronectin*) expression corresponds to *CYP11B2* expression, with presence of negative feedback. **A**, Strong positive linear correlation between *NPNT* and *CYP11B2* expression in 10 pairs of adenomas and their adjacent adrenal, *r*(18)=0.8273; *P*<0.0001. Inset: correlation plot excluding the 2 samples with highest *NPNT* expression, *r*(16)=0.6806; *P*=0.0019. Statistical analysis was conducted by Pearson product-moment correlation. **B**, Representative immunohistochemistry of NPNT and CYP11B2 in corresponding zona glomerulosa (ZG) areas in serial sections of the same adrenal tissue (20× magnification). **C**, Microarray expression of *NPNT* in 7 paired ZG and zona fasciculata (ZF) samples adjacent to a pheochromocytoma and 13 pairs next to an aldosterone-producing adenoma (APA). Bars represent mean expression per group±SEM. Statistical analysis was conducted by 1-way ANOVA followed by Tukey post hoc test. *P<0.05; **P<0.005; ***P<0.0005; NS, not significant. **D**, Negative feedback shown by representative immunohistochemistry of NPNT, in ZG adjacent to pheochromocytoma compared with that adjacent to ZF-like or ZG-like APAs (20× magnification).

Although the ZG is the zone of physiological aldosterone production, CYP11B2 staining is patchy.^22^ The overall analysis of 20 normal adrenal and APA samples revealed a significant positive correlation between *NPNT* and *CYP11B2* encoding aldosterone synthase (*r*=0.82; *P*<0.0001; Figure 2A). At the protein level, areas of *CYP11B2* expression also corresponded consistently with NPNT staining (Figure 2B; Figure S3). We also compared the ZG expression of genes in adrenal adjacent to a pheochromocytoma versus that next to an APA (ie, in a state of aldosterone excess)*. NPNT* was 3.6-fold upregulated in ZG, compared with ZF, when adjacent to a pheochromocytoma, but diminished or absent when adjacent to an APA (Figure 2C). The same observations were made at the protein level (Figure 2D). Similarly, *CYP11B2* was 2-fold upregulated in ZG next to a pheochromocytoma versus next to an APA and 7.8-fold higher on quantitative real-time polymerase chain reaction.

### *NPNT* Drives Aldosterone Production

**Figure 3.**
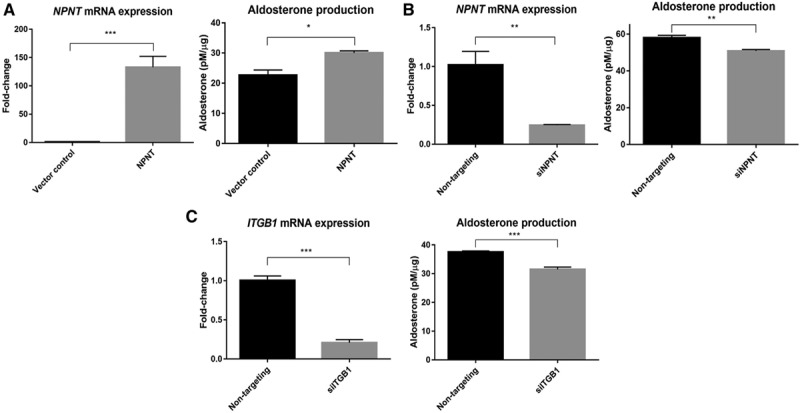
*NPNT* (*nephronectin*) drives aldosterone production, likely through receptor *ITGB1*. **A**, *NPNT* overexpression increases protein-normalized aldosterone production, compared with vector control (n=4). **B**, *NPNT* silencing decreases protein-normalized aldosterone production, compared with nontargeting control (n=4). **C**, *ITGB1* silencing decreases protein-normalized aldosterone production, compared with nontargeting control (n=4). Bars represent mean expression per group±SEM. Statistical analyses were conducted by Student *t* test. *P<0.05; **P<0.005; ***P<0.0005.

*NPNT* overexpression in H295R cells increased aldosterone synthesis compared with control (Figure 3A). Similarly, silencing *NPNT* by >75% reduced hormone production (Figure 3B). NPNT has been previously found to bind strongly and specifically to integrin receptor α8β1,^16^ with ≈100-fold higher affinity compared with other RGD motif-containing proteins such as fibronectin or vitronectin.^27^ In our study, silencing of *ITGB1*, encoding integrin subunit β1, by ≈80%, caused a similar reduction in aldosterone production comparable to silencing of *NPNT* (Figure 3C). This receptor silencing was accompanied by a 3.8-fold increase in *NPNT* mRNA expression (*P*=0.01).

### *NPNT* Is a Wnt Target Gene and Produces Aldosterone via This Pathway

**Figure 4.**
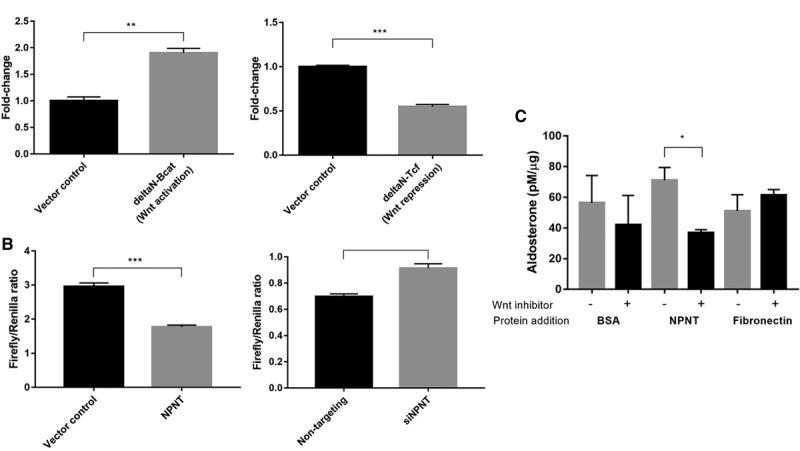
*NPNT* (*nephronectin*) is a Wnt target gene and produces aldosterone via Wnt. **A**, Constitutive Wnt activation (ΔN-Bcat) induces *NPNT* mRNA expression, whereas constitutive Wnt repression (ΔN-TCF4) decreased *NPNT* expression compared with vector control (n=3). **B**, Wnt transcriptional complex T-cell factor/lymphoid enhancer factor (TCF/LEF) activity decreased in *NPNT*-overexpressed and increased in *NPNT*-silenced samples, as measured by firefly/renilla luciferase assay (n=6; n=4). **C**, Wnt inhibitor LGK-974 attenuated the increase in protein-normalized aldosterone production with addition of NPNT protein, compared with negative controls BSA and fibronectin (n=3). Bars represent mean expression per group±SEM. Statistical analyses were conducted by Student *t* test. *P<0.05; **P<0.005; ***P<0.0005.

NPNT was found to be a Wnt/β-catenin target gene in skin, being induced by Wnt activation in the bulge and hair germ cells.^17^ We investigated the influence of Wnt on *NPNT* mRNA expression in H295R cells, using plasmids modulating Wnt signaling. To activate the Wnt canonical pathway, ΔN47 β-catenin, a strong constitutive inducer encoding an N-terminally truncated form of β-catenin resistant to proteolysis,^28^ was expressed. This led to a near doubling of *NPNT* expression levels. Conversely, to inhibit β-catenin–dependent gene transcription, we expressed ΔN-TCF4, a Wnt constitutive repressor, because of an N-terminally truncated, dominant-negative TCF4 protein lacking the β-catenin interaction domain.^29^ Wnt repression caused *NPNT* mRNA levels to halve (Figure 4A). To investigate the potential for negative feedback of NPNT on its own release, TCF/LEF activity was measured after changes in *NPNT* expression. Overexpressing *NPNT* caused a reduction in Wnt transcriptional activity, whereas silencing *NPNT* had the opposite effect (Figure 4B).

In cases where NPNT protein was added to the cell medium, on addition of Wnt inhibitor LGK-974, which blocks Wnt pathways upstream by binding the Wnt chaperone, porcupine, aldosterone production was nearly diminished by half compared with the controls (Figure 4C).

### *NPNT* Is Proadhesive in Normal Adrenal and APA Cells

**Figure 5.**
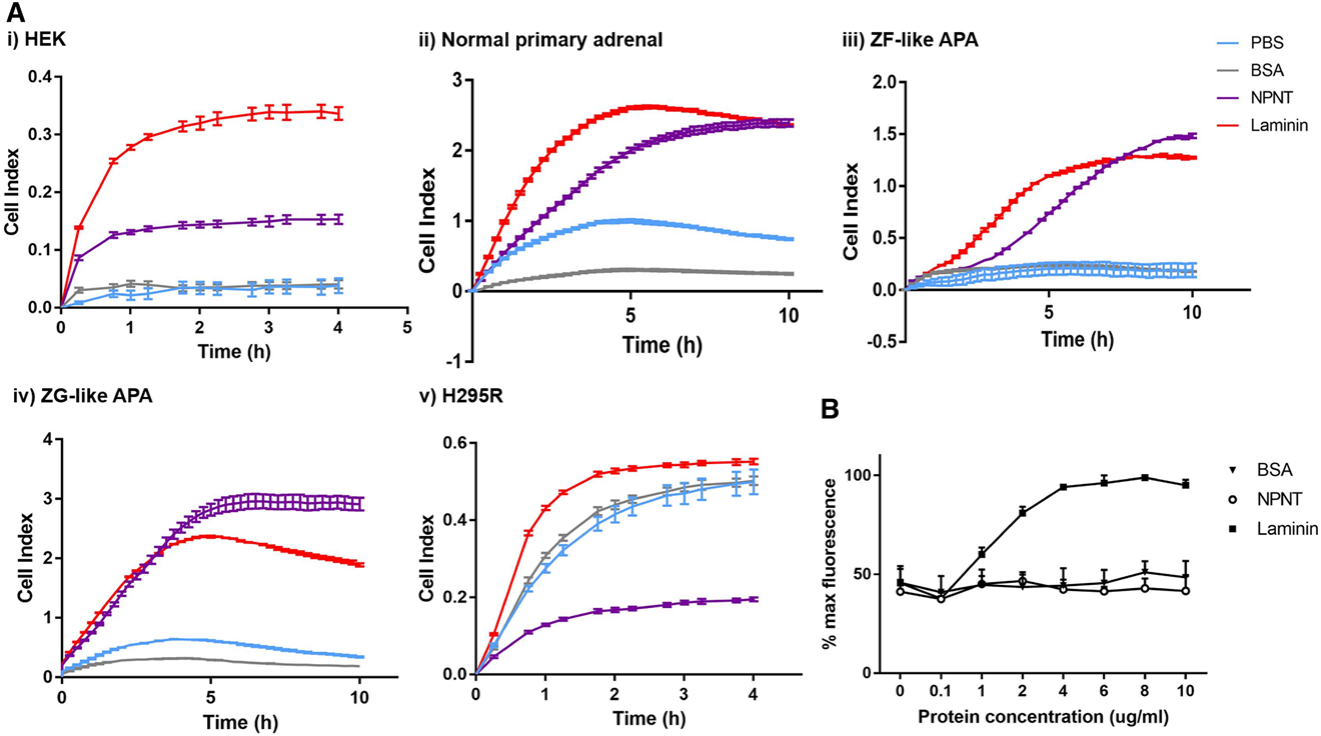
*NPNT* (*nephronectin*) is proadhesive in normal adrenal and aldosterone-producing adenoma (APA) cells, but antiadhesive in H295R. **A**, NPNT is proadhesive in (**i**) HEK, (**ii**) normal primary adrenal, (**iii**) zona fasciculata (ZF)–like APA, and (**iv**) zona glomerulosa (ZG)–like APA, but antiadhesive in (**v**) H295R, as demonstrated by wells precoated with PBS, BSA, NPNT, or laminin, and measured by Xcelligence cell impedance as cell index over time (4 h for cell lines, 10 h for primary adrenal cells; n=2 for ZF-like APA and ZG-like APA, n=4 for the rest). **B**, *NPNT* is antiadhesive in H295R cells, as confirmed by Hoechst fluorescent stain assay measuring % maximum fluorescence as a representation of number of cells adhered to well with increasing concentrations of BSA, *NPNT*, and laminin precoating (n=3 for each protein at each concentration). Bars represent mean values per group±SEM.

NPNT promotes cell adhesion in kidney mesangial cells^30^ and cardiomyocytes.^31^ Cell impedance was recorded as a measure of cell adhesion in wells coated with PBS, negative control BSA, NPNT, or positive control laminin. NPNT was proadhesive in human embryonic kidney cells, normal primary adrenal cells, and cells cultured from ZF-like and ZG-like APAs (Figure 5Ai through Aiv). These findings are consistent with the hypothesized physiological role of NPNT in adrenal cell clustering for aldosterone production.

Intriguingly, NPNT had the opposite effect on H295R cells, with cell index reaching <50% of that in BSA-coated wells even after 4 hours (Figure 5Av). This suggested that NPNT was antiadhesive toward H295R cells, with cells tending to repel from the well surface. To reaffirm this finding, Hoechst stain assays were performed independently, with increasing concentrations of matrix coating on wells. Post-wash, there was a concentration-dependent increase in number of cells remaining (as measured by fluorescence) in positive control laminin–coated wells. However, in NPNT-coated wells, the number of remaining cells showed no significant difference from that in BSA-coated wells at every protein concentration, indicating only background levels of adhesion (Figure 5B).

### NPNT Protects H295R Cells From Apoptosis

**Figure 6.**
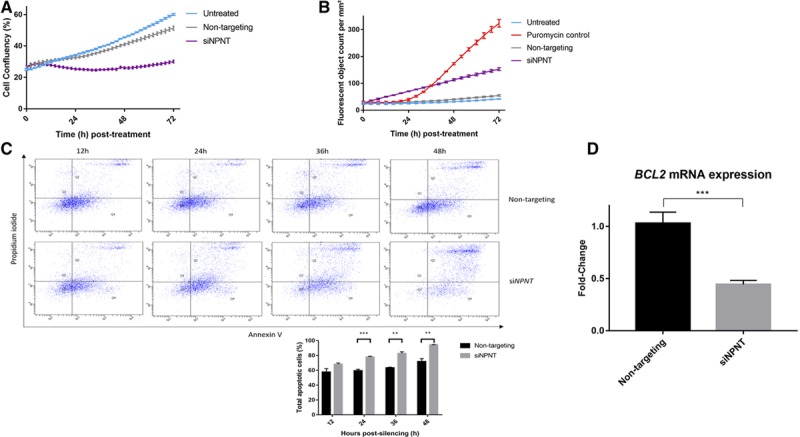
*NPNT* (*nephronectin*) protects H295R cells from apoptosis. **A**, *NPNT* silencing causes cell confluency to remain low over 72 h, as measured by kinetic live-cell imaging in groups that are untreated or treated with nontargeting siRNA (n=3; *P*<0.0001 between si*NPNT* and nontargeting). **B**, *NPNT* silencing drives active cell death, as shown by kinetic measurement of cytotoxicity using YOYO-1 iodide reagent, comparing number of fluorescent (dead) cells in groups that are untreated, treated with positive control puromycin, nontargeting siRNA, or specific siRNA against *NPNT* (n=4; *P*<0.0001 between si*NPNT* and nontargeting). **C**, *NPNT* silencing causes cell death via apoptosis, as shown by flow cytometric analysis using annexin V-APC and propidium iodide double staining in groups that are treated with nontargeting siRNA or specific siRNA against *NPNT*. Quadrant analysis of the gated cells in FL-1 versus FL-2 channels was from 10 000 events. Annexin V+/PI− (lower right quadrant) areas stand for early apoptotic cells, and Annexin V+/PI+ (upper right quadrant) areas stand for late apoptotic or necrotic cells. Graph below shows percentage of total apoptotic cells at 12, 24, 36, and 48 h post-silencing. **D**, *NPNT* silencing causes apoptosis through reduction of BCL2, a prosurvival factor in the intrinsic apoptotic pathway, as shown by mRNA expression in groups that are treated with nontargeting siRNA or specific siRNA against *NPNT* (n=6). Bars represent mean expression per group±SEM. Statistical analyses were conducted by Student *t* test. *P<0.05; **P<0.005; ***P<0.0005.

Cell confluency was 41% in the nontargeting controls compared with 26% in the *NPNT*-silenced samples at 48 hours, as observed using kinetic live-cell imaging (Figure 6A). Kinetic measurement of cytotoxicity via the YOYO-1-iodide reagent revealed >3-fold increase in fluorescent (dead) cells when *NPNT* was silenced (Figure 6B). Annexin V–propidium iodide dual assay was used to monitor H295R cell staining over time. *NPNT*-silenced cells exhibited greater apoptosis over time, whereas samples with nontargeting siRNA consistently showed basal levels of apoptosis (Figure 6C).

A prosurvival factor in the intrinsic apoptotic pathway,^32^ transcription of *BCL2* (*B-cell lymphoma 2*) was greatly suppressed (>56%) in *NPNT*-silenced (si*NPNT*) cells (Figure 6D).

## Discussion

The ZG of human adrenal is unusual among endocrine organs in that few cells produce its signature hormone, aldosterone, and yet there is a high incidence of APA occurrence, which is a common curable cause of hypertension. The ZG has likely evolved primarily to protect mammals from the scarcity of salt that has been the prevailing natural state, including for most of human history. It clearly also has the ability to adapt to chronic salt excess, to which the sparsity of aldosterone synthase is usually attributed,^25^ but perhaps imperfectly, and hence the frequent somatic mutations permitted by high rates of ZG cell migration and renewal. Our discovery of *NPNT*, and its putative roles, in the adrenal may help to explain the link between physiology and pathology.

We first discovered *NPNT* in the adrenal as the most upregulated gene in the smaller ZG-like APAs with higher aldosterone synthetic capacity, harboring mutations of *CACNA1D* or *ATP1A1*, when compared with those with a ZF-like phenotype and mutations of *KCNJ5.* We also noted the exquisite ZG selectivity of its distribution in normal adrenal cortex.^8^ These original findings have been reproduced by Åkerström et al.^33^ Interest in finding a mechanistic link between *NPNT* expression and blood pressure control has also been raised by a large-scale genome-wide association study in which a common single-nucleotide polymorphism in *NPNT* was associated with blood pressure regulation.^34^

The investigations that we now report show that NPNT is not just a marker of ZG cells but plays an essential role in normal adrenal physiology as a periglomerular ECM protein. Coupled with its steroidogenic and proadhesive properties, this is consistent with a physiological role in adrenal cell clustering to form functional aldosterone-producing units in the ZG. Our work provides evidence that a matrix protein can play a role in driving hormone synthesis and, together with the regulation of ZG cell behavior, helps us understand why the ZG may be structured as it is and why tumors that resemble ZG are able to have a higher density of aldosterone production.

The ZG-selective staining suggests that the ECM of which NPNT is a major constituent supports zone-specific cellular behavior. In 2012, Hu et al^20^ made the crucial observation that isolated mouse ZG cells are too hyperpolarized to permit calcium entry; however, ZG cells in an intact adrenal slice generate spontaneous membrane oscillations sufficient for recurrent Ca^2+^ signals, whose periodicity could sustain aldosterone production. Therefore, these findings suggest that the aldosterone-producing ability of adrenal cells requires them to coexist in whole glomeruli, and this process could be regulated by NPNT.

Although the ZG is the zone of physiological aldosterone production, this is not performed by all cells in the area, as evidenced by previous reports of patchy CYP11B2 staining.^22^ But, the mechanism underlying why some ZG cells express *CYP11B2*, whereas others do not, is yet to be determined. Together with the consistent correlation between NPNT and CYP11B2 staining, and evidence showing NPNT increases aldosterone production, we propose that the role of this matrix protein is to cluster ZG cells together to form a functional unit as indicated by its periglomerular staining. This is supported by the recent proposal that Ca^2+^ and Ca^2+^-activated K^+^ channels in ZG cells, when grouped in rosette structures, act as a pacemaker generating the oscillations that regulate aldosterone production.^35^ On NPNT silencing, although the cell-corrected fall in aldosterone appears relatively small (Figure 3B), the absolute change consequent on reduction in both secretion and cell number is more substantial and likely to have considerable impact in intact ZG.

In addition, our own finding of NPNT as an exquisitely selective ZG protein, 3.6-fold upregulated in ZG compared with ZF when adjacent to a pheochromocytoma, but diminished or absent when adjacent to an APA, suggests the disappearance of NPNT when physiological aldosterone secretion is not required. Analogous negative feedback has been reported in ZG adjacent to APA, where both subtypes of 3β-hydroxysteroid dehydrogenase-isomerase (3β-HSD), responsible for synthesizing progesterone from pregnenolone, were found to be suppressed.^36^

Our results also showed that NPNT promotes adhesion in normal adrenal cells and in both subtypes of APAs. This is consistent with previous reports of NPNT expression in hepatitis, inducing the development of granuloma-like cell clusters of hepatocytes.^37^ Cardiomyocytes grown on NPNT not only exhibited increased adhesion but also expressed high amounts of connexin-43 along their intercellular junctions, indicating well-established intercellular communication, and couple electrically with each other resulting in synchronous beating.^31^

The link between increased cell adhesion and aldosterone production lies within the Wnt signaling system. Intracellular Wnt signaling diversifies into several major pathways, including (1) the β-catenin/TCF–LEF pathway (canonical Wnt), which activates nuclear target genes; (2) the planar cell polarity pathway; and (3) the Wnt/Ca^2+^ pathway, with the last 2 being classified as the noncanonical Wnt pathways.^38^ Although *NPNT* expression is itself under control of the canonical pathway, the noncanonical Wnt/planar cell polarity pathway is likely to be involved in mediating NPNT’s control of cell–cell adhesion and the localized assembly of ECM (Figure 4C).^39^ It has been shown that an aberrant planar cell polarity pathway leads to disruption of integrin β1–mediated interactions and, in turn, disorganization of the ECM.^40^ In addition, in line with the proposed role of *NPNT*, integrin β1 expression was reported to be crucial for adhesion of endothelial cells, with its absence causing focal adhesions to become short and disorganized.^41^

Although our experiments concentrated on the physiological roles of *NPNT* in normal adrenal and benign APAs, its antiadhesive and antiapoptotic effect on H295R cells have drawn our attention to a potential role in malignancy. ACCs, although rare, are much more devastating than APAs. NPNT has been shown to confer apoptosis resistance in H295R cells by modulating the expression of prosurvival protein BCL2, whose role is to block caspase activation.^32^ This detachment of cells from the ECM often results in apoptotic cell death known as anoikis.^42^ Excess secretion of ECM components suppresses the physiological induction of anoikis in maintaining normal tissue architecture^43^ and could explain the high levels of *NPNT* expression in adrenocortical carcinoma and immortalized H295R cells (Figure 1B).

However, benign APAs are much commoner than ACCs, and the main translational potential of NPNT may lie in its use as a diagnostic marker in patients with subcentimeter APAs, whose computed tomographic scan and adrenal vein sampling results are inconclusive. Recent publications have reported only 50% concordance between adrenal vein sampling and computed tomography, with >10% of patients aged >50 years lateralizing to opposite sides.^44,45^ Therefore, in vivo measurement of adrenal NPNT could be a more accurate predictor of APA presence and even genotype, as the secreted protein may be measurable in adrenal vein samples routinely collected for unilateral APA diagnoses.

A limitation of the work to date is that the H295R cell line is not a perfect model for native ZG cells or ZG-like APAs, even though H295R cells have proven invaluable in studying mechanisms involved in the physiological regulation of aldosterone production.^46^ Therefore, in the adhesion studies, primary adrenal cell types were also used. In addition, the H295R cell line is already known to harbor a *CTNNB1* mutation^47^ with high levels of NPNT; so, whenever applicable, silencing *NPNT* was prioritized. Furthermore, our work on malignant cells in this study has been performed only on H295R, because of the lack of other human adrenal carcinoma cell lines amenable to transfection. A further limitation is that centripetal migration of adrenocortical cells cannot be studied in human adrenal. Because of the dispersed-cell nature of the experiments which we can currently undertake, the effects of NPNT’s various roles in the adrenal may be underestimated in this study. The full impact of NPNT will become apparent during experiments on intact adrenal with preserved cell–cell contacts. Future work may involve a ZG-selective conditional-knockout of *NPNT*, by crossing a floxed-*NPNT* mouse^48^ with an aldosterone synthase-Cre recombinase mouse.^49^

In conclusion, we have discovered NPNT to be an exquisitely ZG-selective ECM protein in the adrenal. The distribution of NPNT defines the glomeruli anatomically, and its actions explain the critical role of glomerular structure in regulation of aldosterone production and hence blood pressure control. The high levels of NPNT in the smaller aldosterone-dense ZG-like APA subtype, as well as in ACCs, suggest future potential as a diagnostic marker or target for novel therapies.

## Perspectives

Primary aldosteronism is the most common secondary cause of hypertension, of which APAs make up 30% to 50% of cases, with hypertension potentially curable by adrenalectomy. Recently, the discovery of somatic mutations in *CACNA1D/ATP1A1/ATP2B3/CTNNB1* characterize a subtype of small ZG-like APAs that are also histologically and biochemically distinct from the classical large *KCNJ5*-mutant ZF-like APAs. Investigations into transcriptomic differences between the 2 subtypes revealed *NPNT*, encoding the matrix protein nephronectin, to be the most upregulated gene in ZG-like versus ZF-like APAs. Subsequent investigations have shown NPNT not only to be just a biomarker of ZG-type APAs but also to play an important role in normal ZG. Found to be under canonical Wnt control, the distribution and effects of NPNT suggest that it defines the anatomy and function of normal adrenal glomeruli, driving steroidogenesis and adhesion physiologically. In contrast, in immortalized adrenocortical carcinoma cells, NPNT exerts antiadhesive and antiapoptotic effects. Clinical measurement of NPNT in adrenal vein blood may have application in diagnosis of unilateral APAs. Complete cure of hypertension, on removal of *CTNNB1*-mutant APAs, may be predicted through unilateral detection of secreted proteins such as NPNT during adrenal vein sampling. Apart from its potential as a diagnostic marker, the high levels of *NPNT* in the smaller aldosterone-dense ZG-like APA subtype, and in ACCs, make it an attractive molecular target for novel therapies.

## Acknowledgments

We thank the Cambridge NIHR BRC Cell Phenotyping Hub, in particular, Anna Petrunkina Harrison and Simon McCallum and for their advice and support in flow cytometry.

## Sources of Funding

This research was funded by grants from the National Institute for Health Research (NIHR) Senior Investigator award (NF-SI-0512-10052) to M.J. Brown. A.E.D. Teo is supported by the Agency for Science, Technology and Research (A*STAR) Singapore. This study is also supported by Wellcome Trust Translational Medicine and Therapeutics award to M.J. Brown (085686/Z/08/A). S. Garg is supported by the British Heart Foundation (FS/14/75/31134). J. Zhou is supported by the Cambridge Overseas Trust. Additional support was provided by the NIHR Cambridge Biomedical Research Centre (Cardiovascular and Metabolic, and Human Tissue Bank).

## Disclosures

None.

## Supplementary Material

## Novelty and Significance

### What Is New?

- NPNT (nephronectin), selectively expressed in the zona glomerulosa of human adrenal cortex and of aldosterone-producing adenomas arising from this, is shown to have roles in steroidogenesis, cell adhesion, and protection of adrenocortical cells from apoptosis.

### What Is Relevant?

- Aldosterone-producing adenomas are greatly underdiagnosed, one reason being the small size of those appearing to arise in the zona glomerulosa of adrenal cortex. Recognition of their separate identity and of the role of NPNT in steroidogenesis will encourage clinicians to give greater consideration to small adenomas.

### Summary

NPNT is an exquisitely selective, Wnt-driven zona glomerulosa protein, which seems to play an important role in steroidogenesis, by promoting adhesion of ZG cells and preventing apoptosis.
