# Supplementary material for: Physiological and Pathological Roles in Human Adrenal of the Glomeruli-Defining Matrix Protein NPNT (Nephronectin)
Source: Hypertension. 2017 May 10;69(6):1207–16. doi: 10.1161/HYPERTENSIONAHA.117.09156 (PMC5424579; doi:10.1161/HYPERTENSIONAHA.117.09156)
Supplement: Supplementary file 1 [file hyp-69-1207-s001.docx]

**Data Supplement for**

**Physiological and pathological roles in human adrenal of the glomeruli-defining matrix protein nephronectin (NPNT)**

Ada Ee Der Teo^1^, Sumedha Garg^1^, Timothy Isaac Johnson^2^, Wanfeng Zhao^3^, Junhua Zhou^1,4^, Celso Enrique Gomez-Sanchez^5^, Mark Gurnell^6^, Morris Jonathan Brown^1,4*^

***^1^****Clinical Pharmacology Unit, Centre for Clinical Investigation, Addenbrooke’s Hospital, University of Cambridge, Cambridge, UK.*

*^2^MRC Cancer Unit, Hutchison/MRC Research Centre, University of Cambridge, Cambridge, UK.*

*^3^Tissue Bank, Department of Histopathology, Addenbrooke’s Hospital, Cambridge, UK.*

*^4^Centre for Clinical Pharmacology, William Harvey Research Institute, Barts and the London School of Medicine & Dentistry, Queen Mary University of London, London, UK.*

*^5^Division of Endocrinology, Department of Medicine, The University of Mississippi Medical Centre, Jackson, MS, USA; Research and Medicine Services, G.V. (Sonny) Montgomery VA Medical Centre, Jackson, MS, USA*

*^6^Metabolic Research Laboratories, Wellcome Trust-MRC Institute of Metabolic Science, University of Cambridge & NIHR Cambridge Biomedical Research Centre, Addenbrooke's Hospital, Cambridge, UK.*

Short title: Role of nephronectin in adrenal hypertension

**Corresponding author*

*Correspondence to Prof Morris Jonathan Brown, Centre for Clinical Pharmacology, William Harvey Research Institute, Barts and the London School of Medicine & Dentistry, Queen Mary University of London, London, EC1M 6BQ, United Kingdom. Tel: 020 7882 3901, E-mail: morris.brown@qmul.ac.uk*

**Supporting Information**

**Materials and Methods**

**Human subjects**

Post-surgery, APAs and their paired adjacent normal adrenal were identified and macroscopically dissected by histopathologists and separated into three categories for processing: a) snap-frozen in liquid nitrogen and then stored at -70°C for immunohistochemistry or DNA extraction, or b) stored in RNA-later for RNA extraction or c) digested with collagenase for two hours and then placed in Dulbecco's Modified Eagle's Medium (DMEM) /Nutrient F-12 Ham supplemented with 10% fetal bovine serum (FBS), 100 U penicillin, 0.1 mg/ml streptomycin, 0.4 mM L-glutamine and insulin–transferrin–sodium selenite (ITS) media at 37 °C in 5% CO_2_ for cell culture.

The Cambridge database provided 8 ZF-like APAs and 5 ZG-like APAs for microarray analysis between the two tumor subtypes. A further 3 ZF-like APAs and 5 ZG-like APAs were used for subsequent qRT-PCR validation of microarray findings.

**Gene overexpression and silencing**

For *NPNT* overexpression, H295R cells were transfected with GFP-tagged *NPNT* (RG230311, OriGene) or vector control pCMV6-AC-GFP plasmids. For Wnt constitutive activation, H295R cells were transfected with pcDNA ΔN47 β-catenin, a gift from Eric Fearon (Addgene plasmid #19287); for Wnt constitutive repression, transfection was with pcDNA ΔN TCF4, a gift from Bert Vogelstein (Addgene plasmid #16513), or vector control pcDNA3.1 (Invitrogen).

Gene silencing was achieved using short interfering RNA (siRNA) in the form of ON-TARGETplus human NPNT/ITGB1 or ON-TARGETplus Non-Targeting siRNA as negative control. Short interfering RNA (siRNA) sequences were as follows: NPNT 5′-GCAAAUUAUGAGAGUGAGU-3′, Non-targeting 5′-UGGUUUACAUGUCGACUAA-3′. Transient silencing of *ITGB1* was achieved using a pool of four siRNA duplexes that each target a sequence of the *ITGB1* transcripts (ONTARGETplus SMARTpool, Dharmacon). A mixture of four non-targeting siRNA duplexes was used as a negative control (ON-TARGETplus Non-targeting Pool, Dharmacon). The final concentrations of DharmaFECT 1 and the siRNA in each transfection were 0.2% (v/v) and 25nM respectively, in antibiotic-free complete medium. Efficiency of knockdown was evaluated by qRT-PCR 48h post-transfection.

**RNA extraction, reverse transcription and quantitative real-time PCR**

RNA extraction from tissues was carried out by dissecting and homogenising 50-100mg of tissue in a lysis matrix using a homogenizer with 1ml of TRIzol reagent (Life Technologies). To extract RNA from cells, 1x10^5^ cells were stored in RNAlater (Ambion) until 500μL TRizol was added. Total DNA-free RNA was isolated using the PureLink® RNA Mini Kit and the PureLink® DNase Set (Life Technologies) according to manufacturer’s instructions. On column DNase treatment was performed on all RNA samples to elute DNA-free RNA. Concentration of RNA was determined by Nanodrop spectrophotometry prior to reverse transcription. A two-step reverse transcription reaction was performed using AMV reverse transcriptase from Reverse Transcription System (Promega) according to manufacturer’s instructions.

**Immunohistochemistry**

Immunohistochemistry was carried out using the unlabelled antibody enzyme (peroxidase-antiperoxidase [PAP]) method^68^ on fresh frozen human tissue. Sections of fresh frozen adrenals (14 μm) were thaw-mounted onto microscope slides pre-treated with poly-L-lysine to aid adhesion of the tissue section, and stored at -70°C. When required, slides were air-dried overnight at room temperature and fixed by immersion in ice-cold acetone for 10 min. In cases where fresh frozen tissue was unavailable, immunohistochemistry was performed on formalin-fixed, paraffin-embedded adrenal sections (4 μm) using an automated immunostainer with cover tile technology (Bond-III system, Leica Biosystems). Negative controls, in which the primary antibody was omitted, resulted in complete absence of staining. Images were captured using a standard bright-field microscope, a U-TV1-X digital camera and CellD software (Olympus UK).

**Cell confluency and cytotoxicity assay**

Time-lapsed images were obtained using an Incucyte system (Essen BioScience), with ×4 magnification from 16 spots within each well of a 24-well plate every 2h over 72h. Cell confluency was automatically determined from bright-field images at different time-points, using the integrated Incucyte software. YOYO-1 was diluted in cell culture medium and used at a final concentration of 78nM for both experimental and control wells. YOYO-1 fluorescence was measured every 4 h for a period of 72 h, and addition of YOYO-1 alone did not induce cytotoxicity. As YOYO-1 only fluorescently stains the nuclear DNA of cells that have lost plasma membrane integrity, cell death can be monitored in real-time with the Incucyte imaging system and fluorescent signal quantified using the Incucyte FLR object counting algorithm (v2.0), finally represented as the object count per mm^2^. Analysis parameters were as follows: Segmentation- Adaptive 2.0; Background intensity- 20.0 AU; Foreground intensity- 60.0 AU; Manual adjustment- 0.0 AU; Refinement- Edge split v2.0; Edge sensitivity- 0.00; Analysis filter- Area >=50µm^2^.

**Annexin v-propidium iodide dual stain**

Approximately 1 × 10^6^ cells/mL were washed twice with cold PBS, centrifuged at 300 x g, and subsequently re-suspended in 1x binding buffer. 100μL of the solution (1 × 10^5^ cells) was then transferred to a 5ml tube, before 5μL of Annexin V- APC and 5μl PI was added. Cells were vortexed gently and incubated for 15 min in the dark at room temperature, followed by addition of 400μL 1x binding buffer. Finally, samples were analysed by flow cytometry within 1 hour with a Becton-Dickinson FACSCanto II flow cytometer and all data was acquired with the FACSDiva Software. . Trypsinisation of adherent cells led to an overall higher basal apoptotic level across both groups treated with non-targeting siRNA and NPNT-specific siRNA.

**Xcelligence cell impedance measurement and Hoechst stain assay**

Cell adhesion was monitored continually every 15 min using the Xcelligence cell impedance system (RTCA DP Analyzer, Roche) placed in an incubator (humidified atmosphere 5% CO_2_ at 37 °C) for a period of 4 to 10 h depending on cell-type. Real-time cell index was determined using the RTCA DP software.

Cell adhesion was also measured by Hoechst dye quantification of cells remaining on pre-coated wells post-wash. A 96-well clear-bottom black plate was pre-coated with 50μL of purified protein diluted in PBS to varying concentrations and incubated overnight at 37 °C. This solution was then aspirated and non-specific binding blocked with 50μL of 1% BSA in PBS, for 1h at 37 °C. Once again, the solution was aspirated and the wells washed twice with 0.1% BSA in PBS. After trypsinization and in pellet form, cells were washed and centrifuged 3x in serum-free DMEM⁄ F-12 medium. 100μL of the cell suspension (4x10^4^ cells) was transferred into each well and incubated for 2h at 37 °C with 5% CO_2_. The plate was then agitated on a shaker at a fixed rate for 5s at 1000rpm, and non-adherent cells removed by gentle aspiration.

Following that, cells adherent to wells were fixed with 4% formaldehyde for 20 min followed by washing 5x with PBS. These cells were then stained with Hoechst 33342 (5 µg/ml in 0.001% Triton X-100) for 90min at room temperature. After washing with PBS twice more, each well was filled with 100μl of PBS and fluorescence intensities measured using a microplate reader (Synergy HT, Biotek), with an excitation wavelength of 360 nm and an emission wavelength of 460 nm.

**SUPPLEMENTAL FIGURES**

**Table S1. Clinical features of patients and phenotype of tumors involved in the microarray comparing the two tumor subtypes (8 ZF-like APAs, 5 ZG-like APAs).**

**
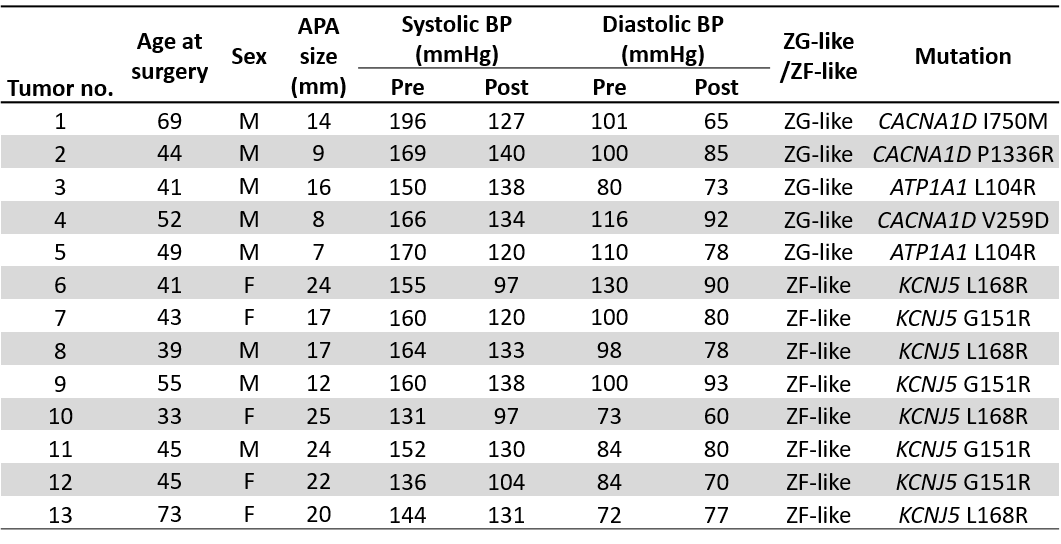
**


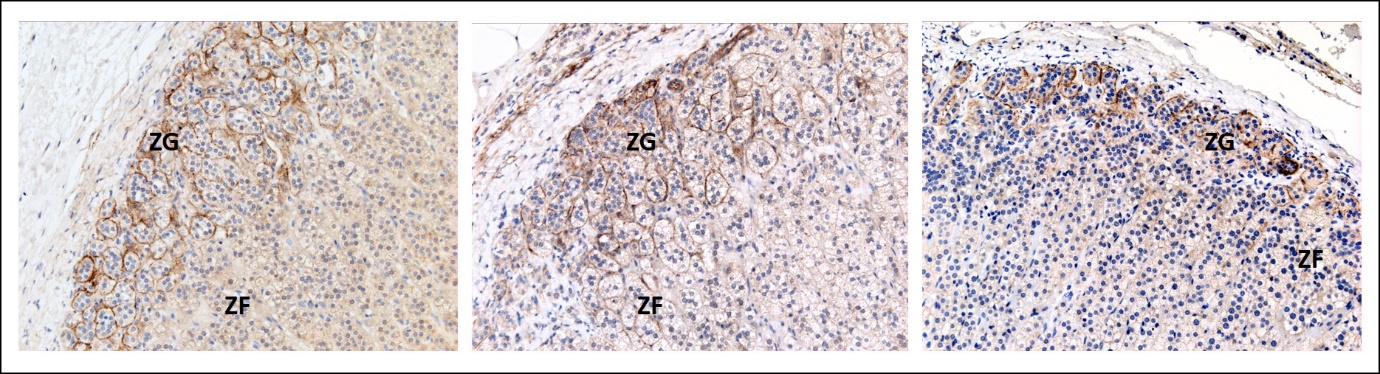


###

### Figure S1. NPNT immunohistochemistry and haematoxylin counterstain in three different formalin-fixed paraffin-embedded (FFPE) human adrenals adjacent to phaeochromocytomas, showing ZG-selective localization and consistent peri-glomerular staining pattern of NPNT.

### (ZG=zona glomerulosa, ZF=zona fasciculata, 20x magnification)


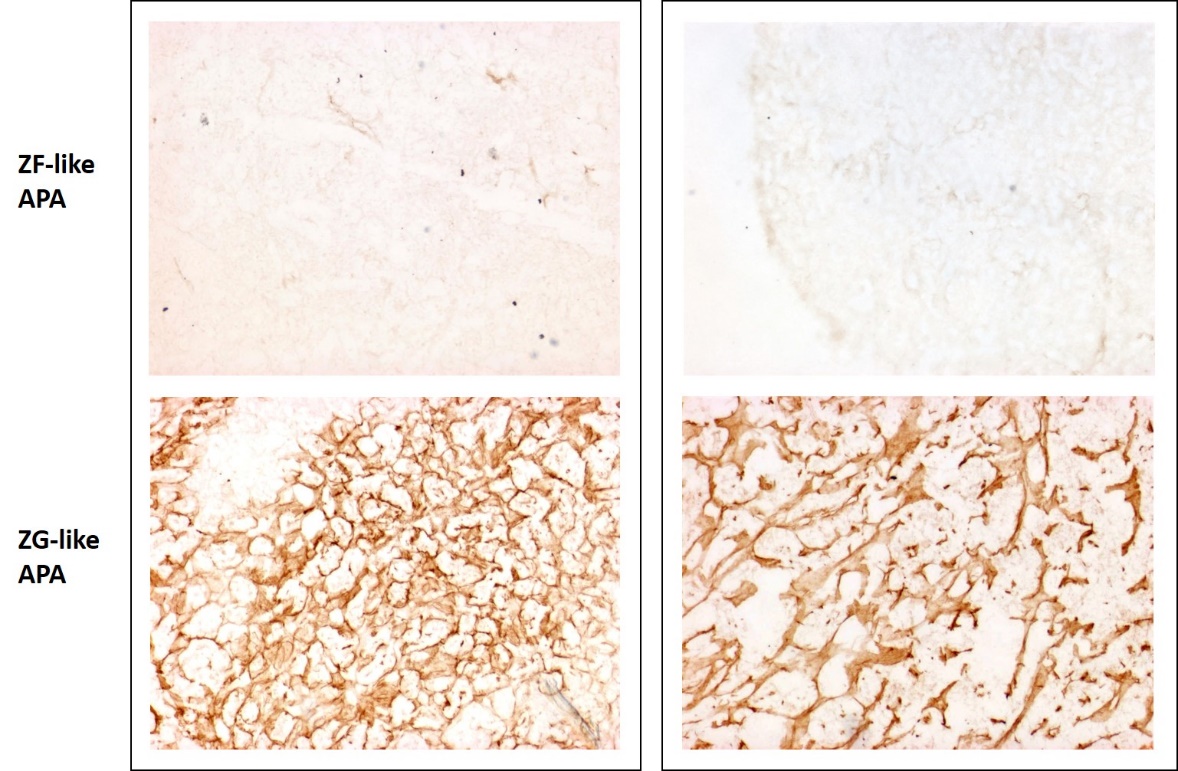


### Figure S2. NPNT immunohistochemistry in ZF-like APA vs ZG-like APA mounted on the same slide, differentiating between the two APA subtypes with negligible NPNT staining in ZF –like APAs

(10x magnification)

###
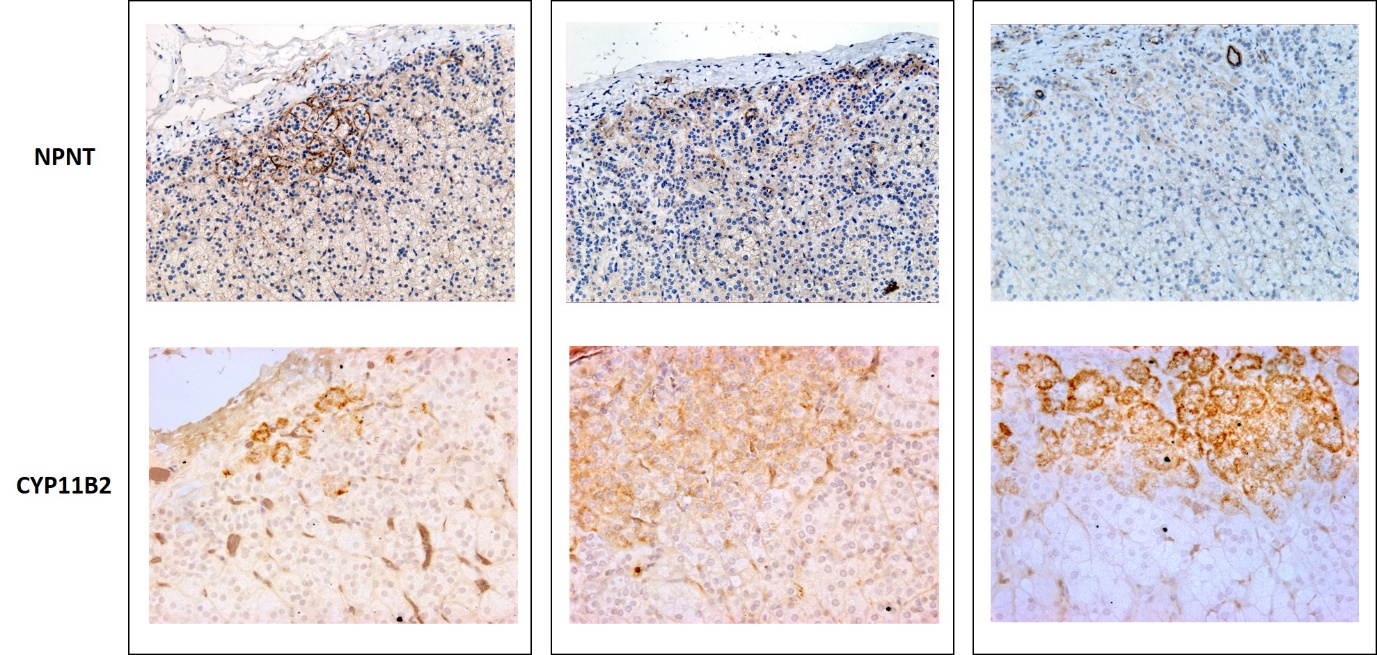


### Figure S3. NPNT expression in aldosterone-producing cell clusters (APCCs) correspond consistently with CYP11B2 expression, as demonstrated by staining of serial sections.

(20x magnification)
